# Case report: Clinical manifestations and genotype analysis of a child with *PTPN11* and *SEC24D* mutations

**DOI:** 10.3389/fped.2022.973920

**Published:** 2022-09-14

**Authors:** Yuqi Miao, Jiahui Chen, Xiaoya Guo, Yu Wei, Xiaozhi Wu, Yanmei Sang, Di Wu

**Affiliations:** ^1^Department of Endocrinology, Genetics and Metabolism, Beijing Children's Hospital, National Center for Children's Health, Capital Medical University, Beijing, China; ^2^Beijing Haidian Hospital, Haidian Section of Peking University Third Hospital, Beijing, China

**Keywords:** LEOPARD syndrome, Cole-Carpenter syndrome-2, *PTPN11*, *SEC24D*, dual mutations, case report

## Abstract

**Background:**

The *PTPN11* gene, located at 12q24. 13, encodes protein tyrosine phosphatase 2C. Mutations in the *PTPN11* gene can lead to various phenotypes, including Noonan syndrome and LEOPARD syndrome. The *SEC24D* gene is located at 4q26 and encodes a component of the COPII complex, and is closely related to endoplasmic reticulum protein transport. Mutations in *SEC24D* can lead to Cole-Carpenter syndrome-2. To date, dual mutations in these two genes have not been reported in the literature.

**Methods:**

We report a patient with short stature and osteogenesis imperfecta as the primary clinical manifestation. Other clinical features were peculiar facial features, deafness, and a history of recurrent fractures. Whole exome sequencing was performed on this patient.

**Results:**

After whole-exome sequencing, three mutations in two genes were identified that induced protein alterations associated with the patient's phenotype. One was a de novo variant c.1403C>T (p.Thr468Met) on exon 12 of the *PTPN11* gene, and the other was a compound heterozygous mutation in the *SEC24D* gene, a novel variant c.2609_2610delGA (p.Arg870Thrfs^*^10) on exon 20 and a reported variant c.938G>A (p.Arg313His) on exon 8.

**Conclusions:**

Concurrent mutations in *PTPN11* and *SEC24D* induced a phenotype that was significantly different from individual mutations in either *PTPN11* or *SEC24D* gene. Personalized genetic analysis and interpretation could help us understand the patient's etiology and hence develop treatments and improve the prognosis of these patients.

## Introduction

There are several reasons for short stature, some of which are syndromes with the main manifestation being short stature, including Turner syndrome, Noonan syndrome, and Silver-Russell syndrome. The primary clinical manifestation of Cole-Carpenter syndrome-2 is osteogenesis, which includes a group of clinical manifestations such as reduced bone mass, increased fragility, craniofacial abnormalities and growth retardation. There are numerous reports on children with short stature and osteogenesis imperfecta, however, patients with dual molecular diagnoses have rarely been reported. Here we describe a Chinese girl with mutations on both *PTPN11* and *SEC24D*. Her unique phenotype is analyzed in detail in this report.

## Case presentation

The proband was an 8-year-old girl who was noticed to be shorter than her classmates for more than 2 years before her parents brought her to our clinic. The patient weighed 3.8 kg at birth and was found to have sensorineural deafness. A cochlear implant was used to restore her hearing. Her communication skills were like any other child her age. However, her pronunciation of certain words was affected. At the age of 2, she suffered a fracture on her right femur twice in 1 year due to two accidental falls. Her leg recovered well after external fixation, and her daily activities were not affected. Her parents noticed her short stature when she was 6 (her height was not recorded). However, they did not take it seriously until 2 years later when her growth seemed to be stunted compared to her school classmates. The patient has a 6-month-old brother who was healthy with no history of fractures. Her parents are of average height with normal body proportions and facial features.

The patient's height and weight were 116 cm (-2.4SD) and 23.5 Kg (-0.8SD). She had normal intelligence. A Cafe-au-lait spot was observed on the skin of the left anterior chest wall, with a few pigmented spots on her face. Her fontanelle has already closed. Abnormal facial features were observed by her physician, characterized by posterior occipital bone convexity, hypertelorism, down slanting palpebral fissures, and a wide and flat nose. Her neck and spine were noticeably short, with a wide, and flat thorax, winged and posteriorly convex scapulae, and bilateral cubitus valgus and genu valgum. She has an overall normal gait. Heart, lung, and abdomen showed no significant abnormalities in physical examinations. Both breast and pubic hair were of Tanner I.

**Figure 1:**
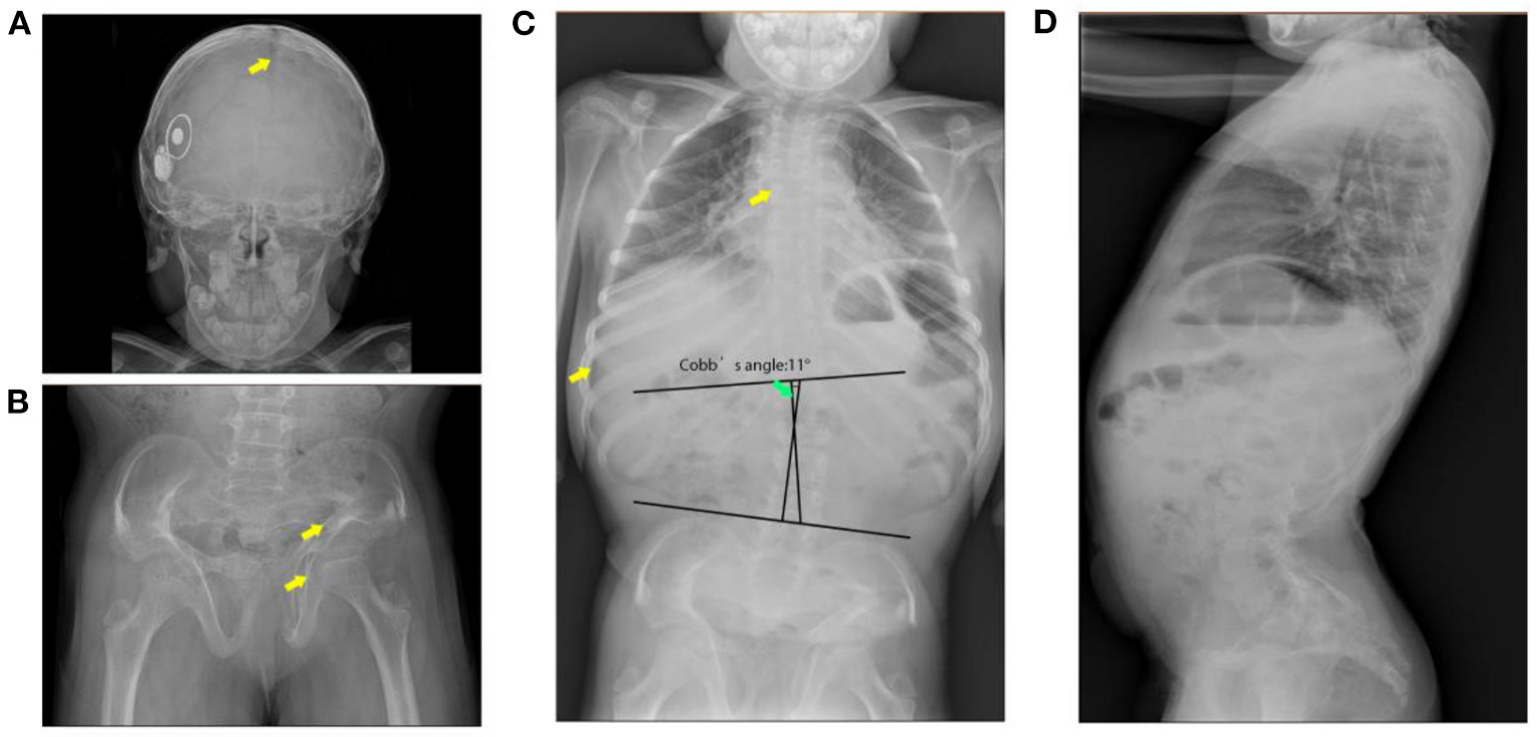
X-ray images of the patient. **(A)** Broad skull with open suture and wormain bones (indicates with the arrow). **(B)** Malformation of the pelvis (indicates with the arrow), low bone density, wide and flat acetabulum (indicates with the arrow), and increased angle of the femoral neck stem. **(C)** Flattened spinal vertebrae (indicates with arrow), thin ribs, and scoliosis. **(D)** Pronounced anterior lumbar convexity.

Skeletal imaging supported the findings of the physical examinations. Broad skull, open sagittal suture, wormain bones, flattened spinal vertebrae, thin ribs, and scoliosis were observed (Cobb's angle: 11°). Her bone age was around 8 years and 10 months old. Abnormal morphology of the pelvis with non-homogeneous bone density and increased angle of the femoral neck stem was observed by pelvis X-ray images (Figure 1). Cardiac ultrasound was not performed. Laboratory examinations for liver and renal function, myocardial enzymes, blood electrolytes, thyroid function, IGF-1, electrocardiogram, bone age, abdominal ultrasound, and pituitary MRI were all normal (Table 1).

**Table 1:** Clinical features of patients with similar molecular diagnosis to our patient.

	**Takeyari et al. (1)**	**Zhang et al. (2)**	**Lin et al. (3)**	**Santoro et al. (4)**	**Carcavilla et al. (5)**	**This report**
Age at diagnosis	15	7	8	50	14	8
Variants in *SEC24D*	p.Arg313His, p.Arg484*	p.Arg313His, p.Pro292Leu	-	-	-	p.Arg313His, p. Arg870Thrfs*10
Variants in *PTPN11*	-	-	p.Thr468Met	p.Thr468Met	p.Thr468Met	p.Thr468Met
Craniofacial dysmorphism	+	-	-	+	+	+
Open anterior fontanelle	+	+	-	-	-	-
Wormian bones	+	-	-	-	-	+
Dentinogenesis imperfecta	-	+	-	-	-	-
Hearing loss	+	-	-	+	-	+
Short neck	-	-	-	-	-	+
Short stature	+	-	+	+	-	+
Scoliosis	+	-	-	+	-	+
Chest deformity	+	-	-	+	+	+
Bone fragility	+	+	-	-	-	+
Cubitus valgus/Genu valgum	-	-	-	-	-	+
Long bone deformity	+	+	-	-	-	+
Lentigines	-	-	+	+	-	-
Heart defect	-	-	+	+	+	unknown
Mental retardation	-	-	+	-	+	-

+, positive; -, negative.

## Methods

Genotype analysis: Informed consent was obtained from the child's guardian. 3 mL of peripheral blood was collected from the child and her parents. Genomic DNA was extracted using the conventional phenol-chlorination method. GenCap^®^ Whole Exon Gene Capture Probe V4.0 (Mackinaw Gene Technology Co. Ltd., China) was used for library construction. Whole-exome sequencing was performed on the MGISEQ-T7 sequencer (UWI Technology Co., Ltd., Shenzhen, Guangdong, China). Reads were mapped to the human genome sequence GRCh37/hg19. Parental Sanger validation of putative pathogenic mutations was performed subsequently.

## Results

**Figure 2:**
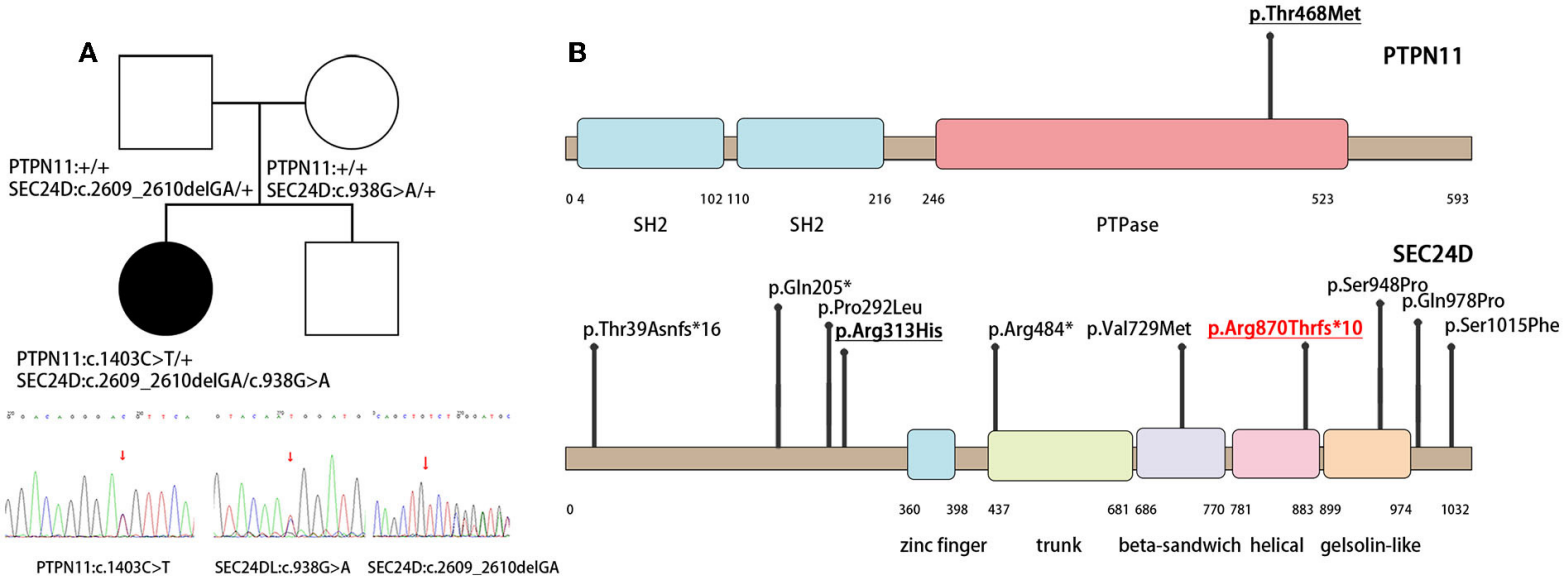
Family of the affected child and illustrations denoting the mutant sites. **(A)** The *PTPN11* and *SEC24D* mutation and the results of the patient's whole-exon sequence. **(B)** Illustrations denoting the mutant sites. Previously found varients in patients with Cole-Carpenter syndrome-2 are depicted in black. The variants found in our patient is underlined and p. Arg870Thrfs*10 in red is the novel variant.

Whole-exome sequencing identified pathogenic variants in both *PTPN11* and *SEC24D* genes. A de novo missense variant c.1403C>T (p.Thr468Met) was identified in exon 12 of the *PTPN11* gene (NM_002834) (Figure 2A), which caused a non-synonymous substitution in the cystine-based protein tyrosine phosphatase domain (Figure 2B). Based on the gnomAD database, this variant has a frequency of 0.000003981 in normal individuals. Protein function prediction software REVEL predicted this variant to be deleterious. Sanger verification showed that neither of the proband's unaffected parents carried this variant. Based on the American College of Medical Genetics and Genomics (ACMG), this variant was classified as a pathogenic mutation. This mutation allele has already been reported in the literature as a hotspot mutation. Clinical phenotypes associated with this variant were LEOPARD syndrome and Noonan syndrome with multiple lentigines (4, 6, 7).

Biallelic variants in the *SEC24D* gene were also identified. One was paternal c.2609_2610delGA (NM_014822) in exon 20 on the helical domain, resulting in a frameshift mutation p. Arg870Thrfs^*^10 (Figure 2A). It was absent in the normal population databases and was classified as pathogenic based on ACMG guidelines. To our knowledge, this variant had not been reported previously. The second was maternal c.938G>A (p.Arg313His) in exon 8 (NM_014822), with a mutation frequency of 0.000641 in normal humans (Figure 2A). It was predicted to be potentially harmful by the protein function prediction software REVEL and was classified as a likely pathogenic variant by ACMG. Compound heterozygotes have been reported in the literature, among which there was a patient with a premature stop codon and the same missense variant of the *SEC24D* gene (Figure 2B) (1, 2).

## Discussion

Among patients with positive molecular diagnoses on whole exome sequencing, 4.9% had two or more genes involved (8). In our study, a heterozygous mutation in *PTPN11* and compound heterozygous mutations in the *SEC24D* gene were identified in the proband.

The *PTPN11* gene, located on 12q24.13, encodes the SHP-2 protein and is widely distributed in heart and skeletal muscle. SHP-2 mediates cell proliferation and serves as a key signaling molecule in the RAS-MAPK kinase pathway (9, 10). Phenotypes of the same *PTPN11* gene may differ between mutated loci, thus, distinctive mutation locus needs to be considered for clinical diagnosis (11). Germline *PTPN11* mutation-related phenotypes include Noonan syndrome, LEOPARD syndrome, and metachondromatosis. Noonan syndrome and LEOPARD syndrome have some overlapping phenotypic features, such as peculiar facial features, sensorineural hearing loss, scoliosis, short stature, and cardiomyopathy. However, multiple lentigines are unique clinical characteristics of LEOPARD syndrome.

Located on 4q26, the *SEC24D* gene encodes a component of the COPII complex, which is involved in protein transportation in the endoplasmic reticulum. Mutations in the *SEC24D* gene result in a reduced outward procollagen transport from the endoplasmic reticulum and dilatation of the endoplasmic reticulum canal, which leads to Cole-Carpenter syndrome-2 (12). Cole-Carpenter syndrome-2 is an autosomal recessive syndrome characterized by abnormal skeletal development due to low bone mass and osteogenesis imperfecta, which include open fontanelle, hydrocephalus, abnormal facial development (e.g., forehead bulge, midface hypoplasia, micrognathia, and ear malformation), and recurrent fractures (1).

Only a few cases of dual mutations have been reported related to the two genes we mentioned above. Martina Caiazza et al. reported a patient with hypertrophic cardiomyopathy who carried a dual mutation in the *PTPN11* and MYBP3 genes (13). To our knowledge, co-mutations of the *SEC24D* gene with other genes have not been reported. The combined effect of the mutant two alleles may explain the difference between the phenotype of this patient and that of a typical patient with a single mutant allele (Table 1).

Short stature is often a parents' focus. However, mixed genetic pathogeny of short stature makes our patient distinctive and relatively poor prognosis. Our patient was of short stature, with a height of 116 cm, and was lower than the third percentile for height for Chinese children of the same age and gender. Patients with Noonan syndrome or LEOPARD syndrome due to mutations in the *PTPN11* gene can develop a short stature (5, 14). For Cole-Carpenter syndrome-2 patients, osteogenesis imperfecta may also result in slower growth and abnormal body proportions (1, 2, 12).

Physical and radiography examinations of the child showed a short neck, scoliosis, valgus scapula, cubitus valgus and genu valgum, and thorax malformation, all of which were consistent with patients with *PTPN11* mutations (15–17). However, the concurrent *SEC24D* gene mutation also played a major role in the skeletal development of this patient, such as wormain bones, pelvis malformation and possible low bone mass. Patients with Cole-Carpenter syndrome-2 are typically characterized by low bone mass, craniofacial abnormalities, various skeletal deformities, and a tendency for repeated fractures (1, 2, 12). The dual gene mutation in this patient could have aggravated the manifestations of bone malformations and low bone mass in the spine, thorax, and pelvis. Repeated fractures also confirm that the *SEC24D* mutation had a significant impact on osteogenesis and bone deformity.

The patient showed abnormal facial features. Orbital hypertelorism, down slanting palpebral fissures, broad and flat nose, and kyphotic occipital bones are similar to the facial features observed in Noonan syndrome and LEOPARD syndrome patients. The wide and deformed skull and wormian bones may be caused by bone abnormalities associated with the *SEC24D* gene mutation. However, *SEC24D* mutant patients may have a more severe phenotype as previously reported, such as a bulged forehead and large, open fontanelle (1, 2).

The number of lentigines in our patient was much lower compared to a typical patient with LEOPARD syndrome. However the site of single nucleotide variation was identified and almost all of the other clinical diagnosis was concordant with LEOPARD syndrome (3, 4), we identified our patient based on gene diagnosis as well. The reason for the absence of lentigines in our patient may be that the disease had not progressed to the certain stage, or the *SEC24D* gene mutation had affected the typical manifestations of the other gene mutation.

Currently, there are no effective etiological based therapies for the LEOPARD syndrome and Cole-Carpenter syndrome-2. Symptomatic treatments should be administered to reverse the impact on their growth and pubertal development. Our patient was found to have hearing loss at an early age, thus, a cochlear implant helped improve her hearing. As a result, her communication was essentially unaffected. Short stature and scoliosis are the biggest concerns for parents of these children. However, some of the patients with *PTPN11* gene mutations were repored to have a relatively poor response to rhGH treatment (18–20). What is perplexing is that patients may develop new scoliosis or accelerate their scoliosis progression after rhGH treatment (21–23). For patients with mild scoliosis (Cobb's Angle <45°), early orthopedic correction has been shown to be beneficial for lower respiratory complications (24). However, due to the probable low bone mass of this patient, spinal fusion could be much more difficult after surgical treatment and may affect her quality of life. A judicious assessment should be performed before resorting to orthopedic surgery.

Even though there were no clinical manifestations of cardiovascular involvement in our patient, the possible risk of cardiovascular abnormalities may be high. Severe obstructive cardiomyopathy is a common cause of sudden death in patients with *PTPN11* mutation (25, 26), thus comprehensive cardiac examinations should be performed for early diagnosis.

## Conclusion

The combined effects of the dual gene mutations observed in our patient led to a phenotype that was not consistent with patients having individual gene mutations. This suggests the importance of comprehensive screening for relevant genomic variations in patients with growth retardation, scoliosis, and facial malformations. For some patients, the reasons for these heterogeneities in the clinical phenotypes may not only be due to the mutation loci but may be due to multiple gene mutations. Hence, proper diagnosis requires a more in-depth, individualized interpretation of the genetic landscape. This will help us understand the driving factors for the clinical manifestations, and hence guide us to formulate appropriate treatment plans.

## Data availability statement

The datasets for this article are not publicly available due to concerns regarding participant/patient anonymity. Requests to access the datasets should be directed to the corresponding author.

## Ethics statement

Written informed consent was obtained from the individual(s), and minor(s)' legal guardian/next of kin, for the publication of any potentially identifiable images or data included in this article.

## Author contributions

The study idea and design were conceived by DW. YM interpreted the whole exon sequencing results and was the major contributors in writing the manuscript. JC analyzed the patient clinical data and gave the further treatment guidance. YS and DW revised the manuscript. All authors participated in the writing of the paper and critical discussions, read, and approved the final manuscript.

## Funding

This article is partly supported by the National Natural Science Foundation of China (82000745).

## Conflict of interest

The authors declare that the research was conducted in the absence of any commercial or financial relationships that could be construed as a potential conflict of interest.

## Publisher's note

All claims expressed in this article are solely those of the authors and do not necessarily represent those of their affiliated organizations, or those of the publisher, the editors and the reviewers. Any product that may be evaluated in this article, or claim that may be made by its manufacturer, is not guaranteed or endorsed by the publisher.
